# Forging just dietary futures: bringing mainstream and critical nutrition into conversation

**DOI:** 10.1007/s10460-021-10275-1

**Published:** 2021-10-27

**Authors:** Carly Nichols, Halie Kampman, Mara van den Bold

**Affiliations:** 1 Department of Geographical and Sustainability Sciences, University of Iowa, 312 Jessup Hall, Iowa City, IA 52242 USA; 2 Environmental Studies, University of California, Santa Cruz, 1156 High St, Santa Cruz, CA 95064 USA; 3 Graduate School of Geography, Clark University, 950 Main Street, Worcester, MA 01610 USA

**Keywords:** Nutrition, Malnutrition, Global public health, Critical food and nutrition studies, Dietary futures

## Abstract

Despite decades of action to reduce global malnutrition, rates of undernutrition remain stubbornly high and rates of overweight, obesity and chronic disease are simultaneously on the rise. Moreover, while volumes of robust research on causes and solutions to malnutrition have been published, and calls for interdisciplinarity are on the rise, researchers taking different epistemological and methodological choices have largely remained disciplinarily siloed. This paper works to open a scholarly conversation between “mainstream” public health nutrition and “critical” nutrition studies. While critical nutrition scholars collectively question aspects of mainstream nutrition approaches, they also chart a different way to approach malnutrition research by focusing on politics, structural conditions, and the diverse ways people make sense of food and malnutrition. In this paper, we highlight the key research agendas and insights within both mainstream and critical nutrition in order to suggest spaces for their potential conversation. We ultimately argue that global public health nutrition interventions might achieve greater success in more equitable ways if they are informed by critical nutrition research. We aim for this intervention to facilitate more substantial crossing of disciplinary boundaries, critical to forging more socially and environmentally just dietary futures in the global South and beyond.

## Introduction

Malnutrition, including undernutrition, micronutrient deficiencies, and overweight and obesity, is the primary cause of poor health globally (Swinburn et al. 2019). Despite decades of research-based policy, programming, and funding, progress on reducing undernutrition remains slow, while rates of overweight and obesity are rising rapidly in many countries (Development Initiatives 2020a, b). Different forms of malnutrition now increasingly co-exist in the same communities, the same households, and even in the same individuals, with concurrent issues such as climate change and COVID-19 posing further challenges to improving nutrition outcomes worldwide (Gillespie 2020; Headey et al. 2020; Swinburn et al. 2019). However, the ways in which social problems, like malnutrition, are framed are never apolitical (Foucault 1984). Indeed, they have significant implications for how related research, policies and interventions are conceptualized, designed, and implemented (Bacchi 2012; Gillespie et al. 2013; Guthman 2014). It is thus imperative to examine the theoretical and epistemological lenses that are used to make visible the problem of malnutrition, and how these lenses shape research questions and intervention designs.

Over the past several decades, traditional or what we refer to in this paper as “mainstream” public health nutrition has amassed a large literature that investigates diverse nutritional challenges by conducting research grounded in theory and methods from economics, public health/epidemiology, and nutrition science (e.g. Black et al. 2008; Ruel and Alderman 2013; Smith and Haddad 2015). Operating from a largely positivist epistemology, this field pinpoints various drivers and consequences of malnutrition, (e.g. Alderman et al. 2006; Smith et al. 2015) and has often relied on experimental evidence from interventions on how to solve the problem (e.g. Bhutta et al. 2013). This work has overwhelmingly shaped research on, and responses to, nutrition challenges worldwide, particularly in the global South.

However, over the past fifteen years, scholars from disciplines such as human geography, anthropology, history, and sociology (among others) have developed a slim but growing body of work that can be referred to as “critical food and nutrition studies” (Biltekoff 2012; Biltekoff et al 2014; Gálvez et al. 2020; Guthman 2014; Hayes-Conroy et al. 2014; Hayes-Conroy and Hayes-Conroy 2016). This work often grounds itself in political economic analysis, while also utilizing post-structural and feminist epistemologies that privilege multiple “truths” and see knowledge as always “partial and situated” (Haraway 1988). Scholars in this field use qualitative, ethnographic, and textual methods to research nutrition inequities and better understand lived experiences of malnutrition, as well as different ways that power operates in food systems and public health nutrition interventions.

While mainstream nutrition scholarship and work emanating from the critical nutrition subfield each contribute important theoretical arguments and empirical evidence to global knowledge about nutrition, these bodies of work have hardly been in conversation with one another. There have been efforts to cross-pollinate between mainstream work on food security and more critical food security scholarship (Leach et al. 2020; Otero et al. 2018), as well as achieve conceptual crossover between mainstream public health nutrition approaches and theoretical frameworks drawn from social theoretical approaches (Nisbett 2019; Walls et al. 2020; Yates-Doerr 2020), but less work has been carried out on how to practically apply such critical nutrition lenses to more mainstream nutrition work.

We argue that global public health nutrition interventions might achieve greater success in more equitable ways if they were more engaged with critical nutrition research. Similarly, critical nutrition work might imagine ways to collaborate across the epistemological fence and translate social theoretical concepts to broader audiences. We highlight the research agendas and insights embedded in each of these bodies of work and suggest spaces for potential conversation. In so doing, we aim to facilitate more substantial crossing of these disciplinary boundaries, as this is critical to forging more socially and environmentally just dietary futures in the global South and beyond. As early career researchers, we have worked extensively within and alongside global health institutions involved in nutrition-related research, policy, and programming in South Asia and West Africa. We are well-versed in critical theory as it pertains to nutrition from our current positions within academia, and we are therefore well-placed to reflect on how these various bodies of work intersect and inform one another. We also acknowledge limitations in our analysis given our own positionalities as white, middle-class, cis-gender women born in high-income countries. Thus, we offer this analysis as a starting point for a conversation that we hope will spur engagement from a wider diversity of researcher, activists, and practitioners engaged in nutrition, broadly conceived.

The structure of this paper is as follows. Section two provides a brief overview of the field as exercised by large institutional donors, research institutes and implementers. Section three outlines insights of the critical nutrition literature that are relevant for global nutrition actors and institutions. Section four provides suggestions for how global nutrition actors—and their funding bodies—might begin to practically integrate these insights. Section five concludes.

## “Mainstream” approaches to public health nutrition

The scale of the malnutrition problem—both in terms of undernutrition as well as rising levels of overweight and obesity—has for decades motivated extensive research, funding, and policy and program development and priorities, with the objective to improve health and wellbeing of communities.^1^

While the scope of this work is vast, much of it has been guided by influential conceptual frameworks focused on multiple facets of malnutrition. These include, among others, identifying and addressing the various drivers of malnutrition (Ruel and Alderman 2013; The Lancet 2013; UNICEF 1990), examining the shift towards diets dominated by processed and staple foods (Popkin 1994), analyzing the “enabling environments” that underpin progress on reducing malnutrition (Gillespie et al. 2013), and assessing the ways in which multiple global challenges reinforce one another through the concept of “syndemics” (Swinburn et al. 2019). Several of these frameworks are worth highlighting.

First, the 1990 UNICEF nutrition framework was developed to identify the various causes of malnutrition with the objective to appropriately design interventions. The framework conceptualizes the causes of malnutrition at the individual (“immediate”), household (“underlying”), and societal (“basic”) level (UNICEF 1990, 1998). “Immediate” includes dietary intake and burden of infectious disease; “underlying” involves food security, maternal and child care practices, and access to health services and safe water and sanitation; and “basic” includes societal factors such as knowledge, resource control, and power and political dynamics. The UNICEF framework has been used throughout much public health nutrition work to design policies and programs and has been important for conceptualizing the multisectoral and intergenerational nature of nutrition (UNICEF 1990, 1998).

Second, conceptualizations of the “nutrition transition”, first developed in 1994 by the scholar of nutrition and obesity, Barry Popkin, have been influential in categorizing countries ‘dietary patterns, and developing interventions aimed to tackle not only undernutrition but also overweight and obesity, along with related non-communicable diseases (Popkin 1994, 2015, 2017; Steyn and Mchiza 2014). This work highlights shifts in dietary patterns from hunter/gatherer diets and those high in fiber and cereals to diets high in fat, sugar, and salt. These shifts have occurred alongside a transition to a more sedentary lifestyle, and economic, demographic, and epidemiological changes such as urbanization, mortality and aging, reduced fertility rates, and reductions in chronic disease.

Third, there has been an increasing focus on improving “enabling environments” for nutrition, including upstream drivers such as political commitment, multisectoral coordination, and financial resources and capacity, to better translate nutrition-relevant policies into programs on the ground (e.g., Gillespie et al. 2015, 2013).

Underpinning this work has been the exponential growth in research and collection of data on causes of and approaches to addressing malnutrition. The 2008 Lancet nutrition series put maternal and child undernutrition in the international limelight, highlighting its long-term deleterious effects on health, identifying interventions that have been proven to reduce undernutrition, and calling for urgent national and international action on the matter. This series also highlighted the importance of focusing on the “first 1000 days”, the period from conception to a child’s second birthday, a period during which poor nutrition can lead to lifelong health and other problems such as diabetes, obesity, chronic diseases, as well as poor school performance and decreased productivity (Bhutta et al. 2013; Black et al. 2008; Bryce et al. 2008; Victora et al. 2008). The 2013 Lancet series on maternal and child nutrition followed with an examination of the effectiveness of interventions focused on addressing undernutrition and micronutrient deficiencies in children and women, their cost and delivery platforms, a review of the nutritional effects of “nutrition-sensitive” approaches, which address underlying determinants of nutrition and health (Ruel and Alderman 2013), and ways to develop politically enabling environments for nutrition (Bhutta et al. 2013; Gillespie et al. 2013; Ruel and Alderman 2013; The Lancet 2013). These Lancet series, alongside other influential publications such as the annual Global Nutrition Report, which highlights the state of nutrition globally (IFPRI 2014, 2015, 2016; Development Initiatives 2017a, b, 2018, 2020a,b), have played a fundamental role in shaping nutrition research, funding, and programming worldwide.

Relatedly, global political momentum on and financial commitment to nutrition has markedly increased over the past decade, with the development of multi-actor networks for resource mobilization (e.g. the 2010 launch of the Scaling Up Nutrition movement), financial pledges to improving nutrition by governments in the global North (2013 and 2021 Nutrition for Growth summits) and donor institutions’ increased focus on nutrition (such as the Bill and Melinda Gates Foundation, USAID Feed the Future, UK’s former Department for International Development).

Lastly, more mainstream approaches have recently seen a shift towards a more explicit emphasis on the structural factors that influence malnutrition, and the interrelated nature of various worldwide pandemics. In a recent Lancet Commission Report (2019), “The Global Syndemic of Obesity, Undernutrition, and Climate Change”, authors point to the reductionist nature of nutrition, and to the importance of acknowledging the systemic and interrelated issues that underlie the global rise in obesity, undernutrition, and climate change, including the “policy influence of powerful commercial actors and inadequate political leadership and governance” (Nugent 2019: 2). There has also been an increased emphasis on researching and addressing nutrition in a more holistic manner, by using mixed-methods approaches and longer study time frames (e.g. Gillespie et al. 2017), as well as analyzing nutrition issues within the context of local power relations, broader political economic dynamics, and other intersecting issues such as climate change (Leach et al. 2020; Salm et al. 2020; Swinburn et al. 2019; Walls et al. 2020). The wealth of data on nutrition outcomes and drivers, as well as the abundance of evidence about “what works” to address proximal determinants of undernutrition (Bhutta et al. 2008) has led to notable impacts on intermediary outcomes, such as women’s empowerment, dietary diversity, and infant and young child feeding practices (e.g. Heckert et al. 2019; Olney et al. 2015; Ruel and Alderman 2013; van den Bold et al. 2015). Still, improvements have been slow, rates of malnutrition remain high, and rates of overweight and obesity are on the rise, presenting communities and households with “double” or even “triple” burdens of malnutrition (Jaacks et al. 2019; Jones et al. 2016; Steyn and Mchiza 2014). Much work thus focuses on scaling up “nutrition-specific” and “nutrition-sensitive” interventions, as well as how to distribute these through different types of delivery platforms, secure adequate funding and capacity, and acquire the necessary political backing and coordination across sectors and administrative levels (Bhutta et al. 2008; Kumar et al. 2018a, b; Meinzen-Dick et al. 2011; Gillespie et al. 2013; Ruel and Alderman 2013).

While the diversity of research within mainstream public health nutrition is vast, there are some broad commonalities across this work that are noteworthy. First, mainstream nutrition largely adheres to postpositivist or positivist approaches to knowledge production (though epistemological commitments or researcher positionality within mainstream public health and nutrition work are rarely made explicit). The methodological toolkit is meant to arrive at certain, generalizable truths through building evidence bases across different populations and geographies. While qualitative approaches with interpretivist analyses have seen a significant increase over the years, these tend to work towards understanding broad thematic trends in the data, rather than using a critical narrative or discursive mode of analysis. Because much of this work is rooted in commitments to “policy relevance” the analysis generally tends to privilege conclusions that are easily translatable to ministries of health, funding agencies, or practitioner networks, and have potential to be “scaled”.

Second, the intervention research that dominates this field is most often underpinned by utilitarian frameworks that focuses on cost-effectiveness and returns to investment rather than rights-based or justice-oriented approaches. This is important given longstanding debates within global health over whether to prioritize universal comprehensive primary care, or focus on providing selective packages of discrete interventions to a targeted population (Farmer et al. 2013).

Third, armed with positivist epistemologies and utilitarian underpinnings, the research and interventions in mainstream nutrition are broadly reformist in aim—attempting to modify behaviors or diets through evidence-based interventions and then securing financing through policy processes to scale-up such programs. While overtly intervening in politics is seen as central to achieving nutritional equity, this is done in a way that often does not necessarily change existing political and economic power arrangements required for achieving more just dietary futures. Rather, it often focuses on framing and communicating the nutrition problem to decision makers, moving nutrition higher up on the political agenda, addressing operational challenges to implementing policy, or modifying the specific packages of nutrition solutions. While positivist epistemologies, utilitarian framings, and policy reform advocacy are all critically important, they simultaneously limit the scope of conversation around more radical visions of nutritionally just futures.

These limitations are highlighted in the next section of the paper where we contrast mainstream approaches with the arguments advanced in critical nutrition. We contend the strength of critical nutrition literature is its commitment to making explicit the messy project of knowledge creation. We show how this may be done through analytically centering the multiple ways that communities value food, nutrition, and health, as well as the structural forms of violence and dispossession that continue to shape nutritional disparities.

## Critical nutrition theory, explained

Critical nutrition, as a subfield, emerged in the past several decades out of an eclectic group of scholars interested in forging more holistic ways of understanding food, diets, and health. Writing from diverse disciplinary perspectives, these scholars collectively critiqued what they understood to be “mainstream nutrition,” and its underlying logics such as nutritionism. According to Gyorgy Scrinis (2013), “nutritionism” describes how nutritional science views food as the mere sum of its constituent nutrients, and how malnutrition thus results from the absence of particular identifiable nutrients. Other scholars have similarly been interested in the ways that Western biomedicine—including nutrition science—has the potential to reduce bodies to indexes of measurable qualities (e.g. weight, height, or hemoglobin levels) (Mol and Law 2004). While the manipulation of nutrients (e.g. through fortification, reformulation, and the creation of functional food) or education on “proper” diets are thus often used as ways to address malnutrition, critical nutrition scholarship points to shortcomings of this approach and worked to theorize and apply less reductive ways to address knowledge and practice.

Thus, the goal of critical nutrition is not simply to critique mainstream nutrition, but to chart a roadmap for how food and nutrition researchers and practitioners might integrate more “critical” perspectives into their work in order to improve impact. Specifically, four critical nutrition guiding principles were published out of a 2009 Canadian workshop aiming to go “Beyond Nutritionism,” and were further developed in a 2013 critical nutrition workshop at UC-Santa Cruz. These guiding principles included (i) acknowledgement that food is more than its constituent nutrients and has multiple meanings plus the power to nourish and health (ii) recognition of human health and disease to be complex and contextual, and (iii) understanding health knowledge to be rooted in socio-cultural and environmental systems (Guthman 2014).

While critical nutrition work initially focused on understanding the social and biocultural implications of nutritional knowledge in high-income countries, scholars have increasingly started to explore these themes in the global South (Burnett et al. 2020; Denham and Gladstone 2020; Ham 2020; Kimura 2013; Nichols 2015, 2020; Yates-Doerr 2015, among others). This research has yielded several key insights that help to contextualize why food and nutrition interventions within marginalized communities residing in the global South often produce only middling levels of impact. We discuss these insights below.^2^

### Multiple values of food

Critical nutrition builds on a vibrant body of critical food scholarship that analytically explores how food has multiple values and political and socio-cultural significance (Appadurai 1981; Coveney 2006; Goodman 1999; Carolan 2011; Probyn 2001). In particular, food is seen as being imbued with both discursive and material power to act on individuals ’identity and their bodily wellbeing in different ways.

*Discursive* power refers to foods having or acquiring socially assigned meanings that define the eater in certain ways. Coveney’s 2006 text was critical in tracing how notions of morality have historically become bound up in dietary practices that change dramatically across time and space. That is, socially defined norms around what is “good food” or what is a “proper diet” influences the ways that people experience, desire, or detest different foods (Coveney 2006). For example, Guthman (2007) has critiqued how fresh fruits and vegetables are associated with notions of enlightened healthfulness, while processed foods like Cheetos may be associated with irresponsibility or slothfulness. This exemplifies how normative food categorizations have long been entangled with classed, racialized and gendered dimensions.

Yet, critical food and nutrition scholars also emphasize that food has *material* power in that eating is a biophysical, “embodied” process that triggers affective states such as pleasure, disgust, or contentment. As a concept, embodied experience suggests that there are a variety of sensorial ways of “nowing” the world beyond cognitive, rational processes (Carolan 2011; Probyn 2001), moving beyond the idea of a “thinking” mind separate from a “feeling” body (Braidotti 2002; Carolan 2011; Grosz 1994; Probyn 2001). Food practices are often shaped by more than political economic factors (e.g. access, purchasing power, household bargaining) or rational ones (e.g. knowledge or discourse), and are deeply tied to sensorial desires and senses of belonging (Hayes-Conroy and Hayes-Conroy 2013; Panelli and Tipa 2009; Richmond et al 2005;). Thus, while mechanisms such as dietary recommendations may influence the social meanings of food (e.g. making them “good” or “bad”) and shape dietary choices, they can never fully control them as food and food desires are experienced in multiple, sometimes conflicting, ways.

For example, in investigating food and nutrition practices among displaced women in Medellin, Colombia, Hayes-Conroy and Sweet (2014) find that “social and environmental imaginaries” of food items shape the way people choose to consume them. Chickens raised and sold in urban contexts were viewed with disgust by women who felt these chickens were given artificial growth hormones and butchered far too quickly for them to develop any flavor. They similarly viewed fish obtained in the city as “inedible” compared to fish in their coastal villages, both because of the taste and also because of the perceived presence of “poison” in them. For these women, nutritionally equivalent animal proteins were markedly different depending on the environmental and social conditions in which they were produced. The authors argue that the women’s narratives revealed more than taste preferences but also their ethical commitments to food produced in environmentally and socially sustainable ways (Hayes Conroy and Sweet 2014: 379).

Other scholars have highlighted how state-led shifts from low-yielding traditional varieties to higher yielding improved seeds also produces different categorizations of “nutritionally equivalent” foods. Ham (2020) finds that the West African staple porridge, *tuo zaafi*, has radically different discursive meanings for her Ghanaian study respondents depending on the type of grain it is prepared from. Lastly, Nichols (2019) addresses the material and discursive power of rice in Central India through detailing how improved varieties of rice may produce higher yields and pathways for upward mobility, but that, for many, these rice varieties are not satiating compared to their traditional (lower yielding) varieties. Moreover, because high-yielding varieties require chemical inputs and adequate irrigation, individuals who prefer traditional crops also value that they are not dependent on markets to purchase seeds or inputs. Expressing desire for traditional varieties rather than the more “aspirational” improved ones is based both on sensorial desire for coarser traditional grains (e.g. the material power of rice), and on political and economic resistance to dependence on agricultural inputs (e.g. it is socially and politically symbolic). Thus, critical nutrition emphasizes that examining the values and perceptions around food goes beyond imperatives for “cultural sensitivity” and, given the complexity of bodies and diets, actually physiologically matters to the nourishment of healthy bodies.

### Biosocial conceptions of health

Just as the production and consumption of food may have multiple meanings, critical nutrition scholars assert that understandings of health are similarly complex. They question whether biomedical understandings of health should hold such vaunted positions in knowledge hierarchies. Rather, they understand bodily ill-ness to be at the nexus of interconnected sociocultural and biological—known as biosocial—processes (Singer and Clair 2003; Singer 1998). Biosocial analyses of nutrition require researchers to investigate people’s diverse explanations for how dietary practices impact bodily health and dis-ease as well as the social, structural, and historical forces that shape these human-food interactions. For example, recent studies on the microbiome link biological gut health with gendered, classed and racialized physical health (Gálvez et al. 2020). In this sense, complex biosocial relationships physically affect the body’s ability to nourish itself. Drawing from feminist epistemologies, biosocial analyses emphasize that even “scientific” knowledge is necessarily partial and “situated,” due to individuals’ positions within particular social, cultural, political and historical contexts (Haraway 1988). These analyses are thus never value-free or devoid of politics and, hence, never entirely objective.

Critical scholars thus question processes of medicalization, which happens when socially produced suffering is cast in medical terms. They caution away from relying solely on objective, universal nutritional standards to assess diets towards using the more expansive concept of “nourishment” or nutritional wellbeing (Burnett et al. 2020; Hayes-Conroy 2016). As a concept, nourishment is less focused on measurable changes in behaviors or bodies (e.g. consumed food groups or biomarkers such as hemoglobin levels), and more concerned with how dietary patterns are implicated in subjective assessments of biophysical and psychosocial wellbeing (Panelli and Tipa 2009). Similarly, dietary disease is often viewed not merely as a product of nutrient imbalances, but stemming from socio-political or environmental factors (e.g. the loss of communal rights over resources) (Richmond et al. 2005).

These theoretical threads coalesce around growing concerns about the “Westernization” of global South or indigenous diets (Popkin 2015), the increased prevalence of diet-related non-communicable diseases like diabetes, and the medicalization of lifestyle and food choices (Kimura et al. 2014). For example, Weaver’s (2015) ethnography documents how women living with diabetes in Delhi, India see their diabetes as a product of social disharmony and “tension” that causes their poor insulin responses, rather than from purely dietary intake and exercise. Mendenhall (2019) looks at how comorbidities like depression and diabetes biologically reinforce each other through inflammatory processes that have sociocultural feedback loops with social stressors and tensions. This work makes clear that “treating” diabetes cannot be done primarily through individualized dietary regimes and insulin schedules because it is as much a product of social distress as it is insulin resistance. Furthermore, categorizing individuals into groups based on such properties might not just introduce new social hierarchies, but also reinforce existing ones based on social factors such as race, class, and gender.

Taking a different approach, Kimura’s (2013) ethnographic works shows how micronutrient malnutrition among Indonesian women has deep roots in social and political economic structures that solutions such as food fortification seek to bypass. Yet, Kimura argues that food fortification, while ostensibly raising micronutrient levels, does little to address the social and political marginalization that women (particularly mothers) face, and thus does little to alleviate their experiences of suffering. Moreover, by “teaching” women how to feed their children, there is further responsibility and stigma attached to mothers, which may inadvertently increase their physical and social suffering. Addressing nutrient levels alone, as well as viewing nutrition as the absence of nutrients rather than shaped by broader social and economic factors, risks fundamentally misdirecting resources.

### Importance of historical and structural context

The focus by critical nutrition scholars on the discursive and material ways that people interact with food and nutrition is always grounded in the histories and structural relations that shape different places (Hayes-Conroy and Hayes-Conroy 2013; Kimura et al. 2014). To understand contemporary health dynamics, critical nutrition scholars place particular emphasis on the power relations that are produced through interconnected political and socio-ecological histories. In so doing, they address structural factors that include (i) social hierarchies (e.g. racism, sexism and colonial legacies), (ii) economic arrangements (e.g. global trade arrangements, liberalization policies, corporate marketing practices), as well as the intersections between the two (Kimura et al. 2014).

While colonial histories have structurally shaped economies, agricultural production, and health systems, they also more insidiously introduced a knowledge and identity politics rooted in ideologies of Western supremacy. Through projects of cultural indoctrination and race science, large populations were coded as “primitive,” and in need of saving (Farmer et al. 2013; Raschke and Cheema 2008). Moreover, critical scholars contend that colonial-era power relations are reproduced in the present day through institutions like the World Bank, transnational corporations, as well as international research and development agencies working among marginalized populations in both the global South (Kimura 2013; McDonell 2015; Yates-Doerr 2015) and the global North (Gálvez et al. 2020; Guthman 2008, 2011). To make sense of this suffering, critical nutrition draws on the concept of “structural violence” (Farmer 1996), referring to the institutional politics that perpetuate notions of Western/white supremacy through an array of economic and political means, from structural adjustment packages with austerity measures to racist housing practices. Moreover, because structural violence is institutionalized, and oftentimes mundane, it animates a narrative that implies that certain people—and places—suffer disproportionately from poor health and nutrition due to some moral failing rather than discriminatory economic policies and political marginalization (Farmer 1996). These historical processes of structural violence have not only shaped population health but remain present in the lives of communities through collective historical trauma and notions of “deservingness” (Burnett et al. 2020; Sotero 2006).

Exemplifying the notion of historical trauma, Burnett (in Gálvez et al. 2020) looks at nutritional violence in Brazil, which she argues emerges from historic anti-blackness and state-sponsored violence. She highlights the intersections between corporate profit-seeking and racism in examining how Black women suffer from the global practices of transnational food conglomerates, which destabilize Black consumption practices and cultures. She focuses on the women who suffer from diseases related to these forms of violence (such as hypertension and diabetes) and analyzes how medical professionals largely attribute these diseases to personal behavior or lifestyle choices. She argues that a focus on individual behaviors ignores the historical processes that enabled transnational food conglomerates to enter local markets, contributing to disintegrating local food cultures. This necessitates attention and care to history and context, as well as a focus on regulatory mechanisms.

While mainstream models of malnutrition pay credence to these contextual determinants, critical nutrition scholars contend that mainstream interventions do not focus sufficient attention or funding dollars on such large-scale social and political reforms, which remain primarily focused on the individual, household, or community (Gálvez et al. 2020). Moreover, critical nutrition approaches aim to go beyond calls for capitalist reform and private industry regulation to create analyses that highlight how white supremacy and colonial logics form the basis of predatory or exploitative capitalist practices in the food system, as well as the often well-intentioned public health interventions designed to address them (e.g. Harris 2016). This obviates the possibility of quick, reform-based solutions to call for deeper interrogations of the ways economic and social power structures intersect to produce nutritional inequities. For example, Guthman (2011) contributes a similar structural critique through her study of scholarship and activism around obesity and food justice in the United States, putting emphasis on how the fundamental structural inequities of race and class intersect as major risk factors for growing body size. Furthermore, “teaching” historically marginalized populations about “proper” diets is insensitive to the historical racist and colonial practices that alienated communities from their traditional foodways. Nutrition counseling and education ungrounded in these histories thus reproduces the dynamics of colonial othering in efforts to “bring good food to others” (Guthman 2008).

## Critical nutrition theory, applied

Having outlined the contours of “mainstream” and “critical” nutrition, we now provide three recommendations meant to facilitate engagement between these literatures and enhance impact on nutrition outcomes.

### Radical equity and inclusion

A central tenet of critical nutrition is the acknowledgment that there are multiple ways of knowing food and dietary health, and that these systems of knowing should be equally valorized (Mudry et al 2014; Hayes-Conroy and Hayes-Conroy 2016). Hence, notions of “participation” within food and nutrition security (FNS) interventions could be rethought in ways that include a wider diversity of voices within decision-making structures. Currently, the standard protocol for making FNS interventions “culturally relevant” or “sensitive” is to conduct formative research using focus groups or participatory methods to better understand cultural beliefs, habits, and traditions. In a sense, this step seeks to translate Western nutritional science in a way that will hopefully be more resonant in different cultural contexts (Dutta 2012). A critical nutrition approach might begin with rigorous engagement to understand community food and nutrition goals. Neither privileging local, contextual knowledge nor Western scientific knowledge, the goal is to understand how multiple epistemologies of food/nutrition could work in concert with each other to achieve locally decided upon goals.

Notions of accountability within FNS interventions would also be challenged in such an approach. Despite widespread recognition that implementing organizations often have more upward accountability to donors than downward accountability to the communities they serve, it appears that little has changed in this dynamic (Ebrahim 2003; Andrews 2014). In this sense, community members may have little real ownership of or deep engagement in development projects. Radical equity and inclusion would involve more than formative research or mid-term process evaluations with course corrections and would necessitate the co-construction of theories of change and project goals with a wide diversity of community voices. Given global enthusiasm around equity in development,^3^ a critical nutrition approach would demand more procedural equity and deep participation by communities in providing feedback or valuable information to shape intervention protocol, and in actual goal setting and project formulation. Here, simple notions of ‘community’ and ‘participation’ would also need to be critically interpreted, with proper attention to making visible and navigating entrenched power hierarchies (Hickey and Mohan 2004). ‘Participation’ is often merely additional labor for already overworked women, and there can be barriers based on literacy, gender, race, class or citizenship that need to be accounted for in program design (Nichols 2020). Thus, labor from community actors requires proper and equivalent compensation to what international or urban staff receive, and there needs to be careful attention to minimizing other social barriers to participation (e.g. caste or citizenship status). This means program staff and evaluation experts need deep knowledge of the sociocultural and political context before inclusionary overtures are rendered (see section “Acknowledging structural history and context”). Our reading of critical nutrition literature suggests that the adoption of such radical inclusionary tactics in international development strategies is critical for developing an evidence base to inform how intellectual humility works in practice, and whether it may lead to interventions with more durable and meaningful impacts.

### Understanding food as symbolic, sensorial, and politically important to perspectives of wellbeing

Opening up elements of project design to the community may challenge dominant forms of monitoring and evaluation. Understanding the multiple values of food and the ways in which it is tied to identity and health could shape monitoring and evaluation in meaningful ways. While mainstream public health institutions produce robust qualitative work examining community perspectives (Nisbett et al. 2017), critical nutrition approaches are distinct in their broader sets of research questions and methodologies (e.g. ethnography, unstructured interviews), offering research participants more latitude to articulate measures important to their wellbeing.

This strong trend towards incorporating qualitative or ethnographic data into program evaluations could be deepened through engaging with critical nutrition perspectives (Boulton et al. 2020). For example, projects could measure community “nourishment” rather than “nutrition” (Burnett et al. 2020; Hayes-Conroy and Hayes-Conroy 2016) and move away from universal standards to assess community well-being. Existing monitoring and evaluation frameworks that measure nourishment rather than nutrition need not abandon normative measures (e.g. body mass index), but rather view them as partial metrics of intervention success. Such frameworks could similarly move beyond a focus on intra-household bargaining to more multi-scalar analyses that unpack how political economies at scales from the local to the global shape resource access and production decisions in interlinked ways. Project indicators could be developed through deep engagement with communities to parse through the elements of food and nutrition that are important to them. This can begin with a decentering of “expert” knowledge on nutrition and a recognition of the ingenuity and innovation marginalized communities have shown in caring for themselves amidst adversity. By actively valuing collective and experiential knowledge as key to forging sustainable food and nutrition futures, a more democratic playing field in developing monitoring and evaluation frameworks could be realized.

### Acknowledging structural context and history

An understanding of the underlying structural context and history of foods is important for examining food as symbolic, sensorial, and politically important to senses of wellbeing. These contexts include historical framing practices, land use patterns, and histories of trade and exchange (Raschke and Cheema 2008; Guthman 2011; Yates-Doerr 2015; Gálvez et al. 2020). As perspectives from critical nutrition push for local relevance, they take into account not just contemporary nuance, but a deep understanding of the historical factors that shape contemporary economic, social and ecological realities.

Critical nutrition scholars are not the first to make the argument for the importance of historical context for equitable interventions. Before the emergence of critical nutrition as a sub-field, civil society organizations such as the international peasant movement La Via Campesina have asserted that structural factors such as globalization and colonialism fundamentally damaged contemporary food systems and health (La Via Campesina 2020). Broader fields of Global Health and Food Studies have consistently documented the need to capture the range of political processes that shape contemporary foodscapes (Dutta 2012; Leach et al. 2020), and notably, select mainstream nutrition approaches have begun to incorporate such perspectives (e.g. Gillespie et al. 2017).

Nutrition interventions in particular stand to benefit from acknowledging structural context and history. A critical nutrition approach would not require completely discarding interventions such as behavior change (for example). Rather, it could enhance these interventions’ success through simultaneously prioritizing multi-scalar analyses and action on structural inequities not just at local levels, but also in national and global underlying causes of the problems at hand to engender longer term solutions both locally and through political action in national and global arenas. Careful study and integration of the interlinked and multi-scalar structural context and history—such as land distribution, social hierarchies, global economic policies, and histories of colonial and neocolonial forces—would precipitate a reorientation of energies. Practically speaking at the project-level, this may require the adjustment or addition of specific personnel to establish, disseminate, and integrate contextual factors at the project design stage.

There is a small but growing literature documenting examples of these three recommendations, which in practice often overlap and interconnect. Narayanan and Rao (2019) describe a participatory program that created spaces for indigenous people in India to collectively analyze structural aspects of community malnutrition (both political economic marginalization and local gender and caste-based power differentials), while also creatively pooling knowledge towards solutions. Similarly, Bezner-Kerr et al. (2019) report on farmer education projects in Malawi and Tanzania, which emphasize nutrition education as part of broader holistic agro-ecological praxis. They developed a curriculum taught by farmers to farmers, which makes explicit links between structural social inequities and nutrition outcomes. Howard (2020) describes a diabetes management program for Indigenous communities in Canada who endured state-sanctioned “residential schools” (in Gálvez et al. 2020). It emphasizes a truthful recognition of the ways in which these schools reinforced symbolically and materially violent relationships to food, and looks toward healing through storytelling, eating together, and centering ideologies of healthful ancestral Indigenous life. These are just a few examples representing a growing body of work.

## Conclusion

This intervention aimed to encourage greater engagement between “mainstream” public health nutrition research and critical nutrition scholarship, in order to stimulate further analysis and conversation about how to forge more just dietary futures.

It is clear that many kernels of critical nutrition perspectives are, in varying degrees, already embedded within the different frameworks that mainstream nutrition uses to guide intervention design and research agendas (e.g. from UNICEF, The Lancet, the nutrition transition). Yet the difference lies in what is emphasized, how resources are allocated, and which people or organizations decide what aspects to focus on. Critical nutrition scholars, while diverse, have central commitments to examining the ways that material and discursive *power* traverse food-body relations as well as the interventions seeking to modify these. In understanding knowledge as a form of power, they analyze the different systems of knowledge that communities use to understand, assess, and assign value to food, health, and bodies that exceed standardized notions (e.g. nutritionism or the medicalization of malnutrition). Furthermore, critically tracing the various political economic and social-ecological histories of *places* and peoples is analytically central to understanding how “the past” is more than context and plays a central role in producing contemporary forms of suffering. In short, critical nutrition argues for more emphasis on the so-called “distal determinants” (e.g. land rights, trade patterns, social services), while also seeking to highlight epistemological diversity in knowing food, bodies, and health that go beyond Western-based scientific or medical ways of knowing.

Insights from critical nutrition could be more fruitfully incorporated into the ways mainstream nutrition experts/researchers use current causal frameworks for malnutrition, and perhaps can aid in producing new ones that reflect multiple systems of knowledge. We recognize that nutritional scientists and their peers continue to produce essential nutrition research and use it to advocate for more political and private sector commitment to reducing nutritional suffering. However, in highlighting critical nutrition’s key insights, we seek to constructively put forth critical perspectives that can deconstruct the persistent patterns of colonialism; bounded and linear thinking as opposed to collectivist thinking; and non-hierarchical solutions. Fundamentally, we contend that progressive action begins with conversation across disciplinary and theoretical boundaries.

## Abbreviations

FNS: Food and Nutrition Security
UC: University of California
UNICEF: United Nations International Children’s Emergency Fund
USAID: United States Agency for International Development

## Footnotes

1 Some of the most influential actors on the research and funding scene include the World Bank, the International Food Policy Research Institute (IFPRI), the Bill and Melinda Gates Foundation, the U.S. Agency for International Development (USAID), the UK Department for International Development (DFID) (now Foreign, Commonwealth & Development Office or FCDO), the United Nations Children’s Fund (UNICEF), and the World Health Organization (WHO), among others.

2 While the majority of examples are located in the Global South, we include similar cases from the settler colonial context in the Global North (e.g. First Nations in Canada).

3 For example, equity is the keynote theme of the 2020 Global Nutrition Report (Development Initiatives 2020a, b).
